# Obstetric Bleeding Study UK (OBS UK): protocol for a stepped wedge cluster randomised trial investigating the clinical and cost-effectiveness of a maternity quality improvement programme to reduce excess bleeding and need for transfusion after childbirth

**DOI:** 10.1136/bmjopen-2026-118723

**Published:** 2026-08-20

**Authors:** Sarah Joanne Kotecha, Claire Potter, Josh Hope-Bell, Nathan S Riddell, Kim Munnery, Olu Onyimadu, Catherine Liberty, Hazel Taylor, Caitlin Dop, Lucy De Lloyd, William Parry-Smith, Julia Townson, Philip Pallmann, Gwenllian Moody, Yvonne Moriarty, Rachel Deere, Christian Barlow, Amrit Dhadda, Amy Elsmore, Seren Willson, Helen Millward, Kate Siddall, Julia Sanders, Simon J Stanworth, Mairead Black, Stavros Petrou, Tanvi Rai, Pauline Slade, Lisa Hinton, Haddy Fye, Ayse Gur Geden, Rachel E Collis, Peter Collins, Sarah Bell

**Affiliations:** 1 Cardiff University Centre for Trials Research, Cardiff, UK; 2 Department of Perioperative Care, Cardiff and Vale University Health Board, Cardiff, UK; 3 Nuffield Department of Primary Care Health Sciences, University of Oxford, Oxford, UK; 4 Department of Primary Care and Mental Health, University of Liverpool, Liverpool, UK; 5 Shrewsbury and Telford Hospital NHS Trust, Shrewsbury, UK; 6 Keele University School of Medicine, Keele, UK; 7 NIHR Midlands Patient Safety Research Collaboration, Birmingham, UK; 8 Leicester Royal Infirmary, Leicester, UK; 9 Cardiff University School of Healthcare Sciences, Cardiff, UK; 10 Department of Medicine, University of Oxford, Oxford, UK; 11 Department of Haematology, Oxford University Hospitals NHS Trust, Oxford, UK; 12 University of Aberdeen Aberdeen Centre for Women’s Health Research, Aberdeen, UK; 13 Cambridge Centre for Frontotemporal Dementia and Related Disorders, Department of Clinical Neurosciences, University of Cambridge, Cambridge, UK; 14 Institute of Education, UCL, London, UK; 15 Department of Anaesthetics, Intensive Care and Pain Medicine, Cardiff and Vale University Health Board, Cardiff, UK; 16 Cardiff University Cardiff Division of Infection and Immunity, Cardiff, UK; 17 Department of Anaesthetics, University Hospital of Wales, Cardiff, UK; 18 Cardiff University School of Medicine, Cardiff, UK

**Keywords:** Electronic Health Records, Blood bank & transfusion medicine, Maternal medicine, Quality Improvement, Clinical Protocols, Randomized Controlled Trial

## Abstract

**Introduction:**

Bleeding during and after childbirth (postpartum haemorrhage, PPH) is the leading cause of severe maternal morbidity in the UK. Between 2017 and 2018, a PPH care bundle termed the Obstetric Bleeding Strategy (OBS) was implemented as a quality improvement project across all Welsh maternity units and improvements in maternal outcomes were observed. The OBS PPH care bundle incorporates assessment of bleeding risk, real-time cumulative quantification of blood loss, escalation of multiprofessional care including more senior staff at defined volumes of blood loss and point-of-care testing of coagulation at 1 L blood loss (or earlier if clinical concern) with targeted blood product transfusion in cases of haemostatic impairment. The Obstetric Bleeding Study UK (OBS UK) will evaluate this intervention in a larger number of maternity units across the UK.

**Methods and analysis:**

OBS UK is a stepped wedge cluster randomised trial designed to test the effectiveness of the OBS intervention compared with usual care on clinical and psychological PPH outcomes after childbirth, evaluate its cost-effectiveness and perform a process evaluation. The study will be capturing data from over 270 000 women and birthing people giving birth in the care of 36 participating maternity units during the 30-month study period. All maternity units will undertake a control period (lasting 3–18 months) during which usual PPH care will be provided, followed by a 9-month implementation period during which the OBS PPH care bundle will be introduced using quality improvement methods and then an OBS UK intervention period (lasting 3–18 months) during which OBS PPH care will be delivered.

The primary outcome is the number of women receiving allogeneic red blood cell transfusion for PPH per 1000 maternities. Secondary outcomes are informed by the core PPH outcome set, psychological and cost-effectiveness measures for women and their partners and a mixed methods process evaluation exploring how the intervention was deployed and possible improvements to inform wider implementation.

**Ethics and dissemination:**

OBS UK will establish whether (and how) the OBS UK PPH care bundle improves outcomes and experiences of women and their partners. Published results will provide evidence to inform PPH maternity care across the UK and internationally. Dissemination of the findings will be made available to members of the public and participants.

**Trial registration number:**

ISRCTN17679951.

**STRENGTHS AND LIMITATIONS OF THIS STUDY**

- Postpartum haemorrhage trial incorporating clinical, psychological, cost-effectiveness outcomes and a mixed methods process evaluation.
- Recruitment of 36 maternity units of variable size, socioeconomic and ethnic composition selected to be representative of the UK population.
- Everyone giving birth in participating maternity units contributes to aggregate data for the primary outcome.
- Conducted in a high-income country so results will have limited generalisability to lower income settings.
- As the whole bundle is being evaluated, further studies will be needed to examine which specific elements are effective.

## Introduction

 Globally, bleeding during and after childbirth (postpartum haemorrhage; PPH) is the leading cause of maternal mortality^1^ and causes 80% of severe maternal morbidity.^2^ The incidence of PPH in many countries is increasing.^3–6^ There has been no improvement in maternal death rates due to PPH in the UK over the last 15 years despite comprehensive guidelines including from the Royal College of Obstetrics and Gynaecology (RCOG).^7 8^

In the UK about 50 000 women (see online supplemental file 1 regarding terminology) per year have severe PPH (>1 L) and of these around 28% require red blood cell (RBC) transfusion.^7–9^ About 5000 women per year in the UK have massive PPH (>2.5 L), with about 320 requiring hysterectomy and 350 admitted to the intensive care unit, being separated from their baby and suffering severe morbidity.^2 10 11^ PPH has long-term psychological consequences for women and their birth partners including post-traumatic stress disorder (PTSD) in 5%–45% of cases and impaired infant bonding.^12–17^

National reviews highlight the need to improve and standardise maternity care across the UK, with recent reports finding wide variations in practice and deficiencies in management in 80% of massive PPH cases.^7 18–21^ Significant inequalities in maternofoetal outcomes have been identified for women of minority ethnicity and socioeconomic deprivation.^7^

It is likely that a standardised, integrated approach is needed to address the multidimensional challenges in PPH management. The E-MOTIVE trial combined quantification of blood loss (QBL) with a bundle of interventions (uterine massage, oxytocic drugs, tranexamic acid, intravenous fluids and examination and escalation of care) to address PPH in low- and middle-income settings.^22^ This study found a 60% reduction in the primary composite outcome of blood loss ≥1 L, laparotomy for PPH or maternal death from PPH (risk ratio [RR] 0.40; 95% CI 0.32 to 0.50).

A PPH care bundle called the Obstetric Bleeding Strategy (OBS) was developed by multiprofessional maternity service stakeholders. The care bundle was implemented as a quality improvement (QI) project in all 12 maternity units in Wales between 2017 and 2018 in an observational pilot study (OBS Cymru).^23 24^ The OBS bundle consists of four interdependent components: risk assessment, QBL during and after all births, escalation of multiprofessional care to more senior staff at defined times and point-of-care testing of coagulation to guide targeted blood component replacement (online supplemental file 2 and online supplemental file 3).

The need for testing was informed by earlier work on fibrinogen, a key component of blood clots, which can fall to critically low levels before other clotting factors during PPH.^25^ Low fibrinogen is associated with placental abruption but may occur unexpectedly.^26^ Infusing coagulation products based on volume of PPH may be inappropriate when coagulation is normal.^11 26^ Fibrinogen >2 g/L is adequate for haemostasis during PPH, but laboratory results take over 1 hour to be available. Validated algorithms for two point-of-care devices, ROTEM and TEG, have been published and updated.^27 28^ Point-of-care coagulation tests provide clinicians with results in minutes, allowing early identification and correction of low fibrinogen levels with concentrated fibrinogen products (fibrinogen concentrate or cryoprecipitate) infused at a time and dose specific to the woman.^29^ This approach was integrated into the OBS Cymru PPH care bundle.

The OBS Cymru intervention was associated with a reduction in massive PPH by 1.10 (95% CI 0.28 to 1.92) per 1000 maternities per year (p= 0.011), but a full evaluation was not possible with the project design.^23^ A robust evaluation of the OBS care bundle requires comparison to usual National Health Service (NHS) care using a rigorous study design incorporating psychological and cost implications and investigating the impact of ethnic and socioeconomic diversity. A process evaluation will inform adoption and mechanisms of impact, ensuring that changes are fully assessed and learning can be applied to other healthcare settings.

### Objectives

#### Primary objectives

To test the effectiveness of the OBS intervention, compared with usual care, on clinical and psychological obstetric bleeding outcomes and evaluate cost-effectiveness.

#### Secondary objectives

To conduct a mixed-methods process evaluation of the OBS intervention, prospectively in participating UK maternity units, and retrospectively in four units in Wales that adopted the OBS intervention during the pilot study.

To conduct a qualitative evaluation of women and birth partners with and without PTSD symptoms after PPH (mental health substudy).

To investigate empiric versus targeted fibrinogen replacement for treatment of acquired fibrinogen deficiency in obstetric haemorrhage (E-Fib substudy).

To collect blood samples from women for further laboratory investigation of mechanisms of coagulopathy (M-Fib substudy, further detailed in separate protocol publication).

This protocol is reported following the Standard Protocol Items: Recommendations for Interventional Trials (SPIRIT) 2025 guidelines.^30^

## Methods and analysis

### Design and setting

The Obstetric Bleeding Study UK (OBS UK) study is a stepped wedge cluster randomised trial involving over 270 000 women at 36 maternity units with nested psychological and cost-effectiveness studies and a process evaluation. The design was selected to allow the intervention to be delivered and evaluated at an organisational level and to incorporate time-based changes in PPH care unrelated to the intervention into the analysis.

**Figure 1:**
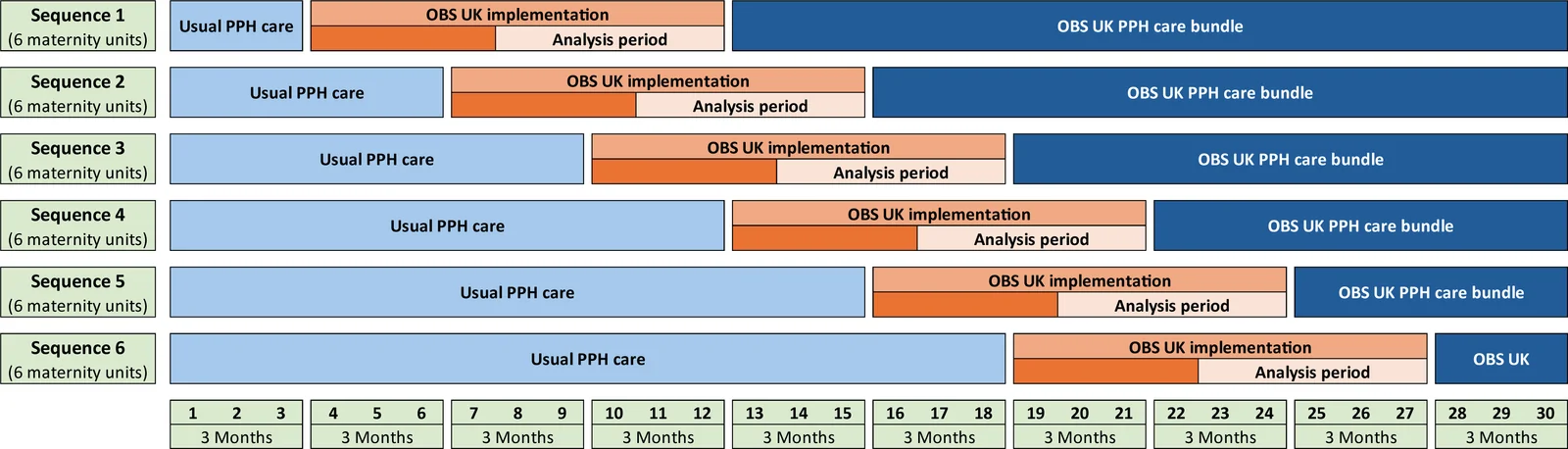
OBS UK stepped wedge cluster randomised trial design. OBS-UK, Obstetric Bleeding Study UK; PPH, postpartum haemorrhage.

Maternity units were randomised to one of six sequences and have collected data during a period of usual PPH care for between 3 and 18 months. They then undertake the 9-month implementation period followed by 3–18 months data collection of OBS UK care (figure 1). Clinical outcome data will be collected and analysed from the usual PPH care period, the OBS UK care period and from months 5 to 9 of the implementation period. The trial started in February 2024 and will finish at the end of January 2027. Sequence 1 was the first maternity units to commence implementation in May 2024 with sequence 6 being the last in August 2025.

#### OBS UK intervention

The OBS UK intervention consists of:

1. The OBS UK PPH care bundle including standardised documentation.
2. Introduced using QI methodology over 9 months.

#### Comparator

Usual UK maternity care.

#### Setting

Sites included 36 NHS maternity units of different sizes and locations serving areas of varied social deprivation and ethnic composition so that the study population approximately reflects the UK population.^31^ A maternity unit includes everyone giving birth in a consultant-led obstetric unit, midwifery-led unit, at home or elsewhere within that service.

### Participants

Maternity unit

#### Inclusion criteria

- NHS maternity units with more than 2000 women giving birth per year.
- Support of the local NHS Trust/Health Board maternity leaders to implement the OBS UK intervention with agreement to support QI time for the local champion team (at least 4 hours/week protected midwifery time and non-clinical time from supporting professional activity for a consultant obstetrician, anaesthetist, and haematologist during the 9-month implementation period).
- One Trust/Health Board may contain several maternity units, if this is the case only one maternity unit per Trust/Board can be included in the study.

#### Exclusion criteria

- Maternity units that have already adopted all aspects of the OBS UK PPH care bundle, including having a point-of-care viscoelastic device on the obstetric unit.

### Individual

#### Inclusion criteria

Primary outcome

- All women giving birth within participating maternity services will have aggregate transfusion data recorded.

Secondary clinical outcomes and E-Fib substudy

- Women experiencing PPH >1.5 L and/or receiving a blood transfusion for PPH will have PPH-specific outcome data collected locally.

Psychology and mental health substudies

- Women who have PPH >1 L and their birth partners (approached in person while an inpatient and offered a £50 voucher on completion of 6-month psychology and economic questionnaires).
- A subsample of participants of diverse ethnicity reporting high and low levels of PTSD symptoms (ie, >33 or >25% of top or <25% of bottom Impact Event Scale score of the sample) at 6 months postnatally will be invited via post or email for interviews.

Economic substudy

- Women who have PPH >1 L and their economic partners.
- Women who have blood loss <500 mL and their economic partners (approached in person while an inpatient and offered a £15 voucher on completion of 6-month questionnaire).

Process evaluation

- Women who have experienced a PPH of >1 L and their birth partners and members of the specialist and wider team at four maternity units included in the pilot study or at any of the six maternity units selected for the process evaluation in OBS UK will be invited for interviews.

E-Fib substudy

- Women infused with a blood coagulation product during a PPH who have a laboratory Clauss fibrinogen <2 g/L up to 120 min before infusion and repeat Clauss fibrinogen up to 60 min after.

#### Exclusion criteria

Secondary clinical outcomes and E-Fib substudy

- Women who have opted out of use of their NHS data for research and/or OBS UK PPH specifically.

Psychology, economic, mental health substudies and process evaluation interviews

- Women who lack capacity to consent, prisoners and those who are <16 years old.
- Women and birth partners who have experienced a stillbirth or neonatal death.

### Trial intervention

**Figure 2:**
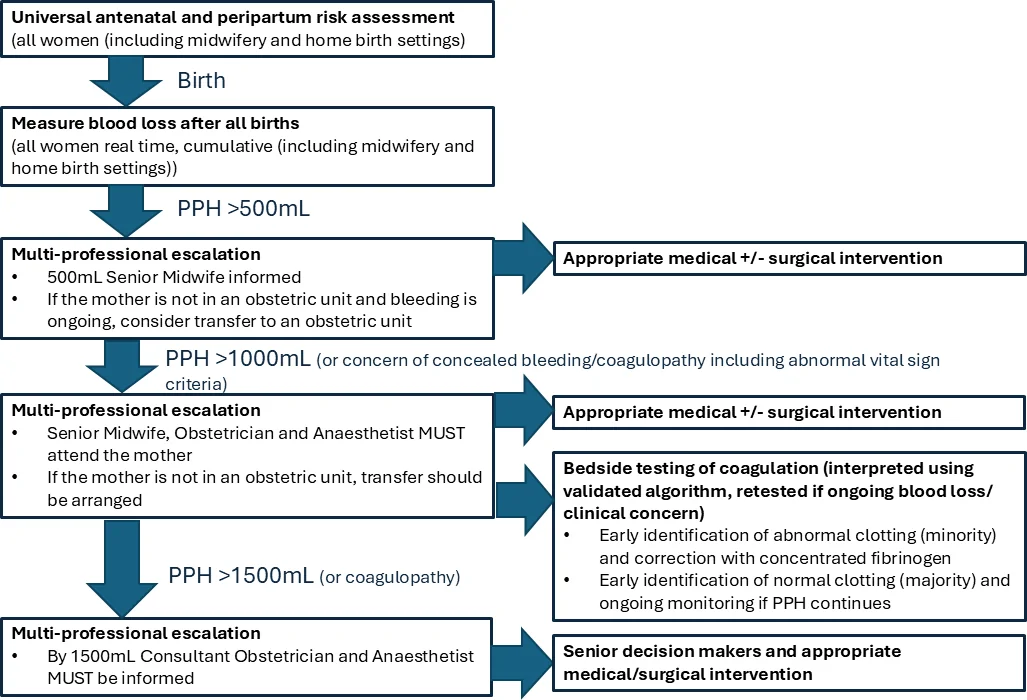
The OBS UK PPH care bundle. OBS-UK, Obstetric Bleeding Study UK; PPH, postpartum haemorrhage.

**Figure 3:**
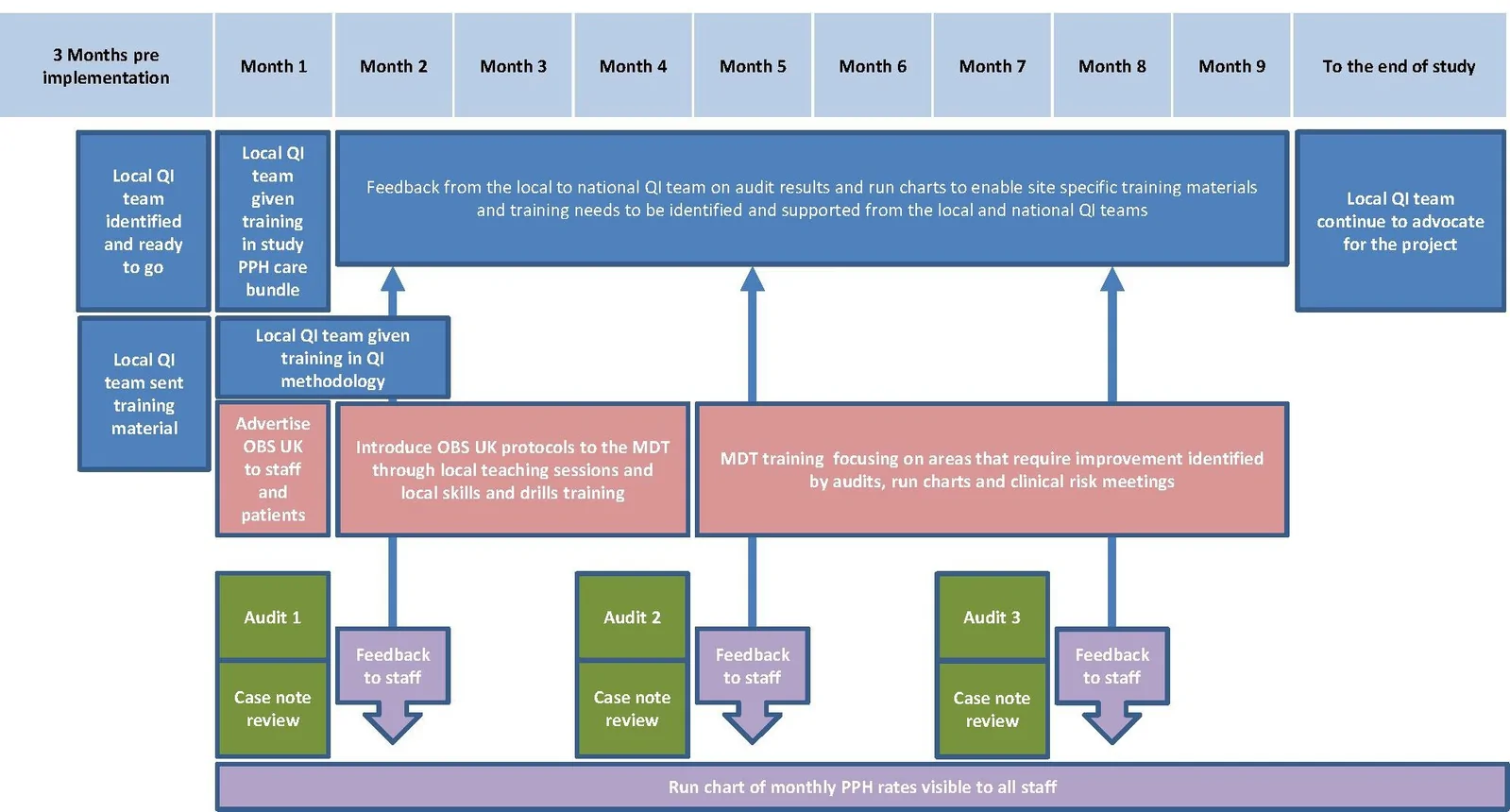
Illustration of the OBS UK 9-month implementation period. At months 1, 4 and 7 of the 9-month implementation period, the local QI team will conduct audits of 30 reasonably consecutive births (all locations, not just obstetric unit births), irrespective of blood loss and undertake case note reviews of 10 reasonably consecutive women who had experienced an obstetric haemorrhage of more than 1L. MDT, multidisciplinary team; OBS-UK, Obstetric Bleeding Study UK; PPH, postpartum haemorrhage; QI, quality improvement.

The OBS UK intervention will be introduced in all maternity areas including midwifery-led and consultant-led units and home births. It consists of:

1. The OBS UK PPH care bundle (figure 2, online supplemental file 2 and online supplemental file 3).
2. Standardised documentation, aligned with current documentation systems.
3. A QI programme to introduce the OBS UK care bundle over the 9-month implementation period (figure 3, online supplemental file 4).

The multiprofessional national QI team will work with the local QI team to support local implementation of the OBS UK care bundle and documentation tool. The local multiprofessional QI team consists of a named QI midwife, obstetrician, anaesthetist and haematologist who will lead local implementation of the care bundle (figure 3, online supplemental files 2 and 4).

### Outcomes

**Figure 4:**
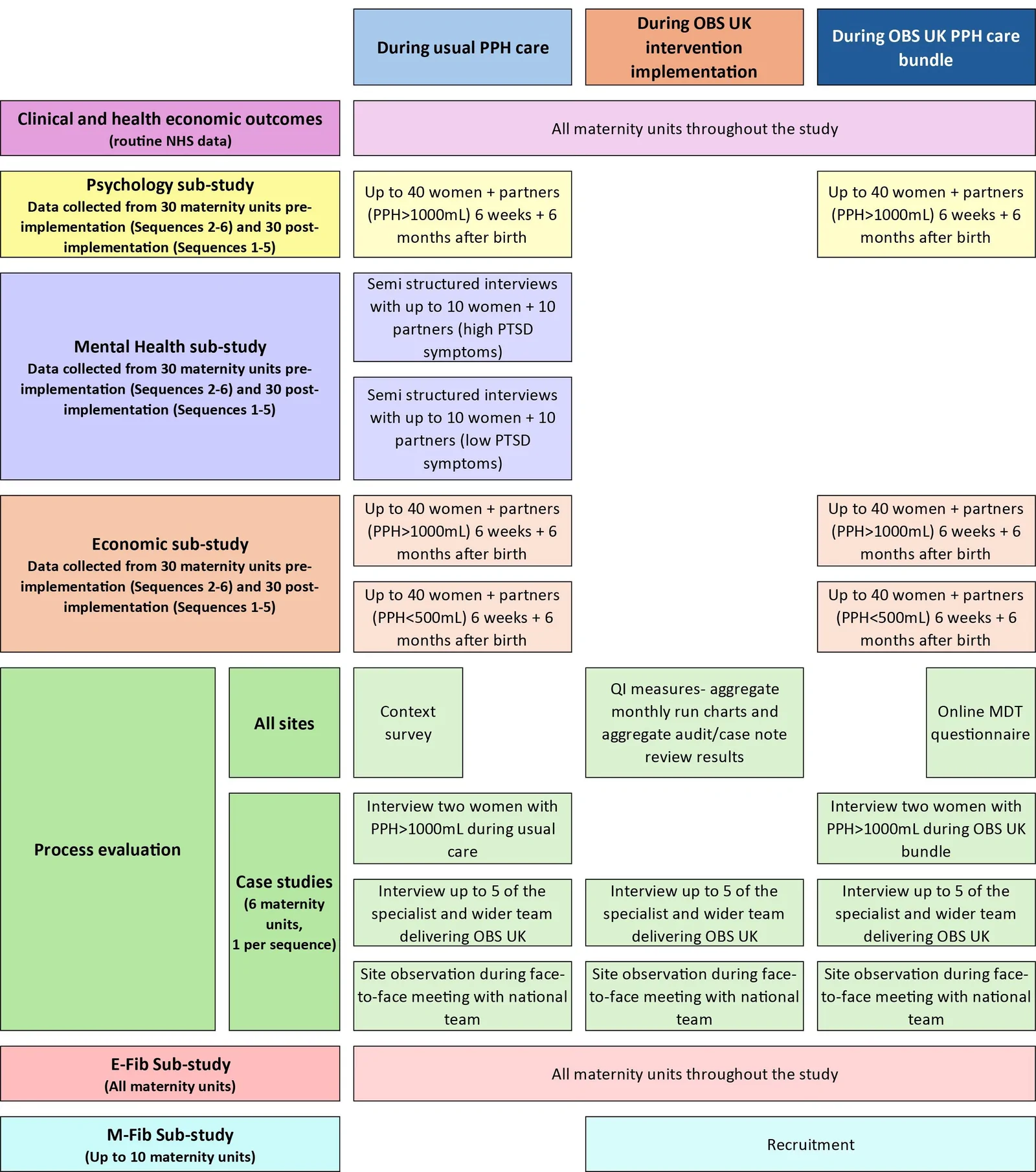
Data collection for clinical outcomes, psychological, mental health and economic substudies and process evaluation. Ethnicity and socioeconomic status of reasonably consecutive women recruited into the psychology and economic substudies at each maternity unit and in each study period (usual care or OBS UK care) will be recorded. MDT, multidisciplinary team; NHS, National Health Service; OBS-UK, Obstetric Bleeding Study UK; PPH, postpartum haemorrhage; PTSD, post-traumatic stress disorder; QI, quality improvement.

See figure 4 for an overview of outcomes and data collection.

#### Primary outcome

The primary outcome is the number of women who receive an allogeneic RBC transfusion for PPH, per 1000 maternities. This will be compared between the usual care period and the OBS UK care period with the addition of months 5 to 9 of the implementation period. RBC transfusions given for obstetric bleeding occurring from 4 hours prior to birth until hospital discharge are included. The local principal investigator will decide whether a transfusion was given for obstetric bleeding.

#### Secondary outcomes

##### Clinical

The following outcomes are reported as number from 4 hours before birth to hospital discharge, per 1000 maternities

1. Blood loss: >1 L, >1.5 L, >2 L, >2.5 L.
2. Blood transfusion: transfused cell salvage and/or allogenic RBCs and the volume.
3. Other blood components/products transfused: transfused coagulation products including type of product (fresh frozen plasma, cryoprecipitate, platelets or fibrinogen concentrate) and number of units or volume.
4. Coagulopathy: fibrinogen levels <2 g/L, rotational thromboelastometry (ROTEM) Fibtem A5 <9 mm or thromboelastography (TEG) citrated functional fibrinogen (CFF) maximum amplitude (MA) (by 10 min) <17 mm, prothrombin time (PT)/ activated partial thromboplastin time (aPTT) >1.5× midpoint of normal reference range and platelet count <75×10^9^.

The following outcomes are reported as number from birth to hospital discharge, per 1000 maternities

1. Hysterectomy: due to PPH.
2. Maternal death: following PPH.
3. Higher level of care: level 2/3, high dependency or intensive care outside of the obstetric unit for complications following PPH.
4. Cardiovascular shock: vasopressor infusion following PPH.
5. Organ dysfunction: organ support following PPH.
6. Haemostatic surgical and radiological interventions: uterine tamponade balloon insertion, uterine brace suture, transfer to theatre or any radiological intervention for PPH.

The following outcomes are reported as stated

1. Neonatal deaths and stillbirths: number, per 1000 maternities.
2. Breastfeeding: time from birth to first feed, type (breastfeeding, formula, mixed), maintenance of breastfeeding including breast milk (only at 6 weeks).
3. Mother and birth partner’s postnatal mental health, acceptability of and satisfaction with intervention, breastfeeding and adverse effects of intervention on mother, baby and birth partner: psychological quantitative study (described below).

##### Additional descriptors

Descriptors will be collected and compared between women with and without severe PPH (defined as >1.5 L bleed) including ethnicity, sickle cell disease, socioeconomic group, body mass index, age, mode of birth, place of birth, parity, induction of labour, cause of PPH.

##### Psychology outcomes

Psychology outcomes collected via questionnaires at 6 weeks (±4 week) and 6 months (±8 weeks) after birth include:

1. Symptoms of PTSD (mother and birth partner) on the Impact of Events Scale Revised^32^ with PPH as a specific event.
2. Symptoms of depression (mother and birth partner) on the Edinburgh Postnatal Depression Score.^33^
3. Mother and baby bonding using the Mothers’ Object Relations Scale.^34^
4. Perspective on whether PPH was psychologically traumatic.
5. Interpersonal factors associated with the experience of the PPH (6 weeks only).
6. Initiation and maintenance of partial/exclusive breastfeeding (mother only).
7. Use of professional support or intervention for psychological symptoms related to the birth experience. What, if any, support has been accessed and participant views on this (mother and birth partner, 6 months only).

##### Mental health outcomes

1. Whether or how experiences of care relate to subsequent levels of PTSD symptoms (6 months).

##### Cost-effectiveness outcomes

The outcomes for the cost-effectiveness analysis (including the EuroQol 5 Dimensions 5 levels (EQ-5D-5L)) are collected via questionnaires at 6 weeks (±4 week) and 6 months (±8 weeks) after birth. Using a unit-specific survey developed by health economic researchers (see Cost Effectiveness Staff Survey online supplemental file 3) and routinely collected NHS England data, a bottom-up micro-costing in a sample of maternity units will identify, measure and value medical, midwifery and support staff costs and consumables associated with risk assessment, monitoring blood loss, testing blood clotting, escalation procedures and follow-on management. Cost apportionment of implementing the intervention at an individual level will account for the size and clinical composition of the maternity unit. These data will be used to assess:

1. Incremental cost per case of allogenic RBC transfusion avoided.
2. Incremental cost per quality-adjusted life-year gained over a lifetime horizon.
3. Length of hospital stay: Time from admission and birth until mother’s discharge.

##### Process evaluation outcomes

1. Prospective data from all maternity units
  - Surveys
    - Maternity unit context survey (study start) and online staff survey at the end of the 9-month implementation period.
  - QI measures
    - Monthly run charts of PPH volume and RBC transfusion throughout implementation and OBS UK care.
    - Audit and case notes review during implementation (1, 4 and 7 months).
  - Assessment of sustainability and/or decay of components of OBS UK care bundle
    - Audit and case notes review in the first month after completion of implementation.
    - Audit and case notes review at the end of study.
  - Individual PPH-specific data
    - Changes in clinical care which impact on the primary outcome (eg, management of antenatal anaemia) may coincide with the introduction of OBS UK care during the study. Therefore, haemoglobin and point-of-care tests will be collected as process measures for those who experience PPH >1.5 L and/or receive a blood transfusion due to obstetric bleeding.
2. Case studies from specific maternity units
  - Ethnographic (on-site observations of training and PPH management, and informal conversations) and qualitative data (in-depth interviews with up to 15 members of the maternity team and with approximately four women (and birth partners, if appropriate) who have experienced PPH) collection from six maternity units (one per sequence) during usual care, implementation and OBS UK care periods and in four maternity units in Wales.

##### E-Fib outcomes

Proportion of women with coagulopathy (fibrinogen level <2 g/L) achieving fibrinogen levels >2 g/L after the blood coagulation product transfusion when given empirical treatment (usual UK care) versus targeted concentrated fibrinogen transfusion (OBS UK care).

### Data analysis and statistics

#### Sample size

##### Primary outcome

The anticipated reduction in RBC transfusion from 27.5 to 18.0 per 1000 maternities is based on the effect size seen in the OBS Cymru pilot study and recently published UK PPH transfusion rates.^9 23^ The average unit size was anticipated to be about 3000 maternities per year.^35^ A stepped wedge cluster randomised trial (using maternity units as clusters) recruiting over 30 months, with 6 sequences and an estimated average of 750 maternities per 3-month period per unit, requires 5 units to be randomised to each sequence. The total of 30 units gives 93% power to detect the above effect size, based on a two-sided type I error rate of 5% and assuming a ‘discrete-time decay’ correlation structure with intra-class correlation of 0.002 (based on previous audit data and similar studies,^36^ and cluster auto-correlation of 0.80 while assuming no data from the 9-month implementation phase will be included in the analysis). With data from implementation months 5–9 included in the analysis (reflecting an assumption that transition to OBS UK care will be underway after 4 months) we will have 90% power to detect a reduction in the primary outcome from 27.5 to 22.0 RBC transfusions per 1000 maternities. The computations were performed using the Shiny CRT Calculator.^37^ One additional unit per sequence will be randomised (total of 36 units) to account for potential drop-out. Based on a more recent estimate of average maternity unit size of 3500 maternities per year, this corresponds to approximately 270 000 maternities to be included in the analysis. The primary outcome is collected monthly from each site using aggregate data and we do not anticipate missingness within the dataset.

##### Psychology, cost-effectiveness and mental health substudies

Data will be collected from 30 maternity units preimplementation (sequences 2–6) and 30 postimplementation (sequences 1–5). The sample size calculation is based on recruitment of mothers experiencing PPH and requires 40 to be recruited per maternity unit over a 6-month period preimplementation and the same number again postimplementation (allowing for 75% attrition, as observed during the early stages of this study) at 6-month follow-up. This will provide ≥80% power for a medium effect size (Cohen’s delta of 0.5) based on a two-sided alpha of 5% and assuming an approximate normal distribution of outcome data. Maternity units will recruit a comparator group of mothers who have experienced a PPH and their birthing partners.

The same women and partners will be recruited for the psychology and cost-effectiveness substudies. The cost-effectiveness study will also recruit up to 40+40 women and economic partners (EP) who did not experience a PPH from before and after the intervention.

At least 10 women and birth partners who report high levels of PTSD symptoms and at least 10 birth partners reporting low levels of PTSD symptoms recruited from the preimplementation psychology substudy will be interviewed in the mental health substudy.

##### Process evaluation

The process evaluation includes 3 days of ethnographic observations during each phase of the OBS UK study. Retrospective case studies involving interviews with 5–6 staff and 2–3 women who experienced a PPH >1 L will be conducted in four Welsh OBS Cymru maternity units.^38^ Prospective case studies in six sites (one per sequence) will include interviews with 12–15 maternity team members sampled across the three study phases and approximately four women who have had a PPH >1 L and their birth partners from the usual care and OBS UK care phases.

##### E-Fib substudy

Based on data from our recent publication and theoretical modelling,^26 29^ we anticipate that the proportion of women with fibrinogen level <2 g/L during obstetric haemorrhage who achieve a fibrinogen level of >2 g/L after the first cycle of coagulation product transfusion will increase from 30% with usual treatment to 70% with OBS UK treatment. To detect an effect of this magnitude with 90% power with a two-sided 5% type I error rate, 62 women are required (based on a χ^2^ test). To account for up to 22.5% missing, the sample size is inflated to 80 women (40 receiving usual treatment and 40 OBS UK treatment).

### Randomisation

Maternity units were randomised all at once by the trial statistician to one of six sequences before the start of the study using R. A covariate-constrained randomisation procedure was used to ensure that half the maternity units in each sequence were allocated to ROTEM and half to TEG. This approach was used to prevent severe imbalances with respect to size of maternity unit (births per year) both between maternity units using different types of devices within the same sequence and between sequences. The final allocation was selected at random after discarding the worst 10% of possible allocations with the highest imbalance.

### Main analysis

Clinical and sociodemographic characteristics of women in participating maternity units as well as unit characteristics will be summarised descriptively and graphically.

The primary analysis will be intention-to-treat, meaning that all participants will be analysed as receiving usual care or OBS UK care (including data from women in months 5–9 of the implementation period) according to the randomisation status of their obstetric unit, regardless of adherence to the intervention. The primary estimand will use a treatment policy strategy for intercurrent events. It will use a logistic mixed-effects regression model with cluster (maternity unit) and cluster-time interaction fitted as random effects, fixed effects for time period (to capture secular trends) and exposure status as well as fixed effects for the stratification variables (unit size and point-of-care device) to estimate the OR of receiving RBC transfusion for PPH between OBS UK care and usual care, presented as a point estimate with a two-sided 95% CI and p value. If the estimate favours OBS UK care and the 95% CI excludes one, effectiveness of the intervention will be concluded. Similar analyses will be performed for the secondary clinical and psychological outcomes with linear instead of logistic models used for continuous outcomes for which at least approximate normality can be assumed.

Sensitivity analyses may consider alternative correlation structures, secular trends that differ between (randomised groups of) units, and intervention effect heterogeneity across time and/or (randomised groups of) units.^39 40^ In a further sensitivity analysis, a marginal mean model will be fitted using generalised estimating equations with robust SEs instead of mixed-effects regression, allowing a more population-focused interpretation.^41^

Subgroup analyses will explore the impact of ethnicity and/or socioeconomic factors and other aspects of demographic diversity on the intervention effect, by adding interaction terms with intervention to the analysis model.

If some units implement individual elements of the OBS care bundle prior to entering the 9-month implementation period, or if individual units fail to implement the intervention during the 9-month period, this will be modelled in a separate sensitivity analysis. No unit will have adopted the point-of-care tests of coagulation or used QI methods for implementation of the entire OBS UK care bundle prior to the 9-month implementation period. The variation in prior adoption and/or partial implementation of individual elements will also be measured and considered in the process evaluation. The process evaluation will include exploring the role of sociodemographic and ethnic differences in staff and patient populations and build on the principles of equality, diversity and inclusion (EDI) in the analysis. A combination of Normalisation Process Theory and the Consolidated Framework for Implementation Research will be used to analyse data.^42 43^ Currently, neither framework attends explicitly to intersectionality nor EDI concerns, but these theories will be developed in this regard.

A statistical analysis plan will be agreed and signed off prior to database lock. Reporting of results will follow the Consolidated Standards of Reporting Trials (CONSORT) statement and its extension to stepped wedge cluster randomised designs.^44^

## Ethics and dissemination

### Ethical, legal and regulatory aspects

Ethical approval was granted by Northwest–Greater Manchester Central Research Ethics Committee (REC 23/NW/0242). The intervention is delivered at a maternity unit level, and aggregate primary outcome data are reported for all women giving birth in the care of participating maternity services.

Electronic health records (EHR) will be retrieved under section 251 approval from the Confidentiality Advisory Group in England and Public Benefit and Privacy Panel for Health and Social Care (HSC-PBPP) in Scotland. Routine EHR currently cannot be requested in Northern Ireland and so pseudonymised PPH specific data will be collected and no linkage performed. Information about the study is displayed throughout participating maternity units, and online, including how to opt out of use of EHR for research (England) or the OBS UK study (England and Scotland). In Scotland women who have a PPH of >1.5 L and/or receive a blood transfusion for PPH are given a leaflet about the trial explaining how to opt out. Routine EHR will be linked to individual-level PPH specific data to develop a de-identified PPH research dataset for each nation. Data sources include Hospital Episode Statistics, Maternity Services, Emergency Services, Community Services and from Scottish equivalents. This will allow in-depth analysis of clinical outcomes, demographic and obstetric variables, economic and psychological outcomes and process measures.

Informed consent is obtained for the psychological, economic and process evaluation questionnaires, surveys and interviews (see Patient Consent Form online supplemental file 2 for an example). Amendments to trial documents including the protocol will be reviewed by the sponsor and REC. The Centre for Trials Research (CTR) will issue the site with the latest version of the documents as soon as they become available.

### Data management

Full details of the data management plan are available from: https://fundingawards.nihr.ac.uk/award/NIHR152057. This includes details of how data and confidentiality will be protected in line with NHS and University guidelines.

### Data availability

Anonymised OBS UK data will be made available on reasonable request to the study management group.

### Monitoring

The clinical trial risk assessment has been used to determine the intensity and focus of central and on-site monitoring activity in the OBS UK trial. Moderate monitoring levels will be employed (online supplemental file 5).

### Protocol deviation

Failure to fully implement or abandoning one of the components of the care bundle will not be regarded as a protocol deviation, however, if a site decides that an element of the bundle cannot be implemented at all this would be a protocol deviation and they will be withdrawn from the study. Implementation will be graded and considered as a sensitivity analysis. Appropriate obstetric care is permitted as per local guidance. Any additional trials at participating sites related to PPH or anaemia management must be discussed with CIs prior to consideration of inclusion.

A maternity unit starting to use a point-of-care device (ROTEM or TEG) located on the unit during the usual care period would make the unit ineligible and so would be considered a protocol deviation. If this were to happen then the unit would not be withdrawn and would be included in the planned analysis, using a treatment policy strategy.

### Safety considerations

Since the components of the care bundle are recommended and used throughout the UK^8^ (although inconsistently), no adverse events are anticipated as a unique consequence of participation in the trial, and no expedited reporting of adverse events is planned. Maternal death and critical care admissions will be collected for all participants and monitored by the combined trial steering committee and Independent Data Monitoring and Ethics Committee.

If a woman receiving fibrinogen concentrate has an adverse drug reaction, the Medicines and Healthcare products Regulatory Agency (MHRA) will be informed using the Yellow Card scheme as per usual clinical practice, but these will not be reported as adverse events for the OBS UK trial. CSL Behring will be informed via email as described in the Pharmacovigilance Agreement if the MHRA is informed of any adverse drug reactions associated with Riastap supplied for study use.

All participants will be recruited at NHS sites and therefore the NHS indemnity scheme/NHS professional indemnity will apply with respect to claims arising from harm to participants at site management organisations.

### Patient and public involvement

Two women with lived experience of PPH from ethnic minority backgrounds are co-investigators. The research team, in collaboration with Egality Health, has facilitated workshops with organisations from under-represented communities to discuss patient-centred outcomes, advise on ethical issues (including consent and management of personal data) and develop communication materials to tackle unconscious bias, inform communities of the study and seek their endorsement. The workshops have also informed the establishment of an Advisory Group for the study who meet at least 6 monthly. The Advisory Group have influenced the psychological questionnaires, all public-facing materials and reviewed the plain English summary.

### Dissemination

The results of this study will impact policy and practice on a national and international level. To support rapid review of national guidelines and practice, results will be communicated to RCOG, Royal College of Anaesthetists, Royal College of Midwives and National Institute for Health and Care Excellence. The dissemination strategy and materials will be co-developed with our Patient and Public Involvement Advisory Group. Study outcomes will be published in peer-reviewed journals and presented at conferences and forums targeting key stakeholders including local maternity services.

## Discussion

The OBS UK trial includes a large nationally representative sample, robust stepped wedge cluster randomised design and comprehensive evaluation of clinical, psychological and economic outcomes within routine clinical settings. Some limitations should be considered. First, the OBS UK care bundle is being evaluated as a whole, meaning further studies will be needed to examine the individual components of the intervention. Additionally, the study is being conducted in a high-income country so generalisability to countries with lower economic status will be limited. Lastly, the use of more accurate and objective measurement methods may increase the number of PPH events detected compared with clinical estimation performed during usual care. Any observed increase in PPH incidence should therefore be interpreted in the context of improved ascertainment rather than a true increase in haemorrhage.

Overall, the OBS UK trial will generate comprehensive evidence to inform both clinical practice and future research aimed at improving outcomes for women experiencing PPH.

## Supplementary material

10.1136/bmjopen-2026-118723 online supplemental file 1

10.1136/bmjopen-2026-118723 online supplemental file 2

10.1136/bmjopen-2026-118723 online supplemental file 3
