# Supplementary material for: Obstetric Bleeding Study UK (OBS UK): protocol for a stepped wedge cluster randomised trial investigating the clinical and cost-effectiveness of a maternity quality improvement programme to reduce excess bleeding and need for transfusion after childbirth
Source: BMJ Open. 2026 Aug 20;16(8):e118723. doi: 10.1136/bmjopen-2026-118723 (PMC13504888; doi:10.1136/bmjopen-2026-118723)
Supplement: Supplementary file 1 [file bmjopen-16-8-s002.pdf]

OBS UK: Clinical and cost-effectiveness of a maternity quality improvement programme to reduce excess bleeding and need for transfusion after childbirth: the Obstetric Bleeding Study UK

Site ID | Patient ID

CONSENT FORM

(Please **initial** each box and sign in full at the bottom of the page)

1. I confirm that I have read and understood the Information Sheet for OBS UK PIS economic and psychology mothers and birthing people Bleed (version 1.6, dated 18.02.2025) for the OBS UK study. I have had the opportunity to consider the information, ask questions and have had these answered satisfactorily.
2. I understand that my participation is voluntary and that I am free to withdraw at any time, without giving any reason, and without my normal medical care or legal rights being affected.
3. I understand that the research team may be required to look in my medical notes. I give permission for them to do this.
4. I understand that a member of the research team will need to contact me by telephone, email, text message or post to complete some questionnaires. I give permission for them to do so.
5. I understand that the information I provide will be held confidentially for up to 15 years before being destroyed. I understand that if I withdraw from the study then data collected up to the time I withdraw will be retained. I also understand that no information I provide will be given to anyone outside this particular research study.
6. I understand that information about me may be looked at by Cardiff University, The University of Liverpool and The University of Oxford supervising the study for audit purposes. I give permission for these individuals to have access to these records.
7. I understand that information collected about me that is held and maintained by NHS Digital and other central UK NHS bodies, collected from medical records and other health-related records may be looked at by the research team and responsible practitioners during the trial. I give permission for these individuals to have access to these records and for them to be used in this research on the understanding that all information will remain confidential.
8. I understand that information collected about me (including name and address) will be held at the Centre for Trials Research, Cardiff University according to the 2018 General Data Protection Regulation (GDPR) (EU 2016/679). I understand that this information will be kept strictly confidential and that no personal information will be used in the study report or publications.
9. I agree to take part in the above trial.

Optional Consent

1. I agree for any data collected to be shared with other researchers for use in future research. This data will not include any identifiable information.

Yes No

Name of participant (please print):_____________________________________ Signed:___________Date:___/___/___

Name of person taking consent (please print):__________________________ Signed:___________Date:___/___/___

This study/project is funded by the NIHR HSDR programme (NIHR152057). The views expressed are those of the author(s) and not necessarily those of the NIHR or the Department of Health and Social Care.

Local Researcher:
