# Supplementary material for: Obstetric Bleeding Study UK (OBS UK): protocol for a stepped wedge cluster randomised trial investigating the clinical and cost-effectiveness of a maternity quality improvement programme to reduce excess bleeding and need for transfusion after childbirth
Source: BMJ Open. 2026 Aug 20;16(8):e118723. doi: 10.1136/bmjopen-2026-118723 (PMC13504888; doi:10.1136/bmjopen-2026-118723)
Supplement: Supplementary file 2 [file bmjopen-16-8-s003.pdf]

OBS UK Care Bundle Costing Survey

This survey is designed to capture information from clinical staff on the resources required to deliver the OBS UK bundle. It focuses on staff time, roles, and any additional activities or shifts introduced as a result of implementing the bundle. These data will be used to inform site-specific cost estimates, support micro-costing of bundle components, and ensure that the OBS UK economic evaluation reflects the practical realities of implementation across different settings. We understand the answers to some questions will be highly variable depending on the clinical situation. However, we are interested in what happens in practice, so responses should reflect usual or most common practice. We expect that the local PI will complete this survey after consulting with their multidisciplinary colleagues.

* Required

Background Information

1. Hospital name and NHS Trust *

- ◯ Ashford & St Peter's Hospital NHS Foundation Trust
- ◯ Barts Health NHS Trust
- ◯ Bolton NHS Foundation Trust
- ◯ Calderdale & Huddersfield NHS Foundation Trust
- ◯ Chesterfield Royal Hospital NHS Foundation Trust
- ◯ Dartford & Gravesham NHS Trust
- ◯ East Lancashire Hospitals NHS Trust
- ◯ East Sussex Healthcare NHS Trust
- ◯ Guy's and St Thomas' NHS Foundation Trust
- ◯ Hampshire Hospitals NHS Foundation Trust
- ◯ Homerton Healthcare NHS Foundation Trust
- ◯ Kettering General Hospital NHS Foundation Trust
- ◯ Kings College Hospital NHS Foundation Trust - Princess Royal University Hospital
- ◯ Maidstone & Tunbridge Wells NHS Trust
- ◯ Mid & South Essex NHS Foundation Trust
- ◯ Mid Yorkshire Hospitals NHS Trust
- ◯ Milton Keynes University Hospital NHS Foundation Trust
- ◯ NHS Grampian
- ◯ NHS Lanarkshire
- ◯ NHS Tayside
- ◯ North Tees and Hartlepool NHS Foundation Trust
- ◯ Northampton General Hospital NHS Trust
- ◯ Northumbria Healthcare NHS Foundation Trust
- ◯ Portsmouth Hospitals University NHS Trust
- ◯ Royal Surrey County Hospital NHS Foundation Trust
- ◯ Salisbury NHS Foundation Trust
- ◯ South Eastern Health & Social Care Trust
- ◯ South Tees Hospitals NHS Foundation Trust
- ◯ South Tyneside & Sunderland NHS Foundation Trust
- ◯ South Warwickshire NHS Foundation Trust

- ◯ Surrey & Sussex Healthcare NHS Trust
- ◯ The Rotherham NHS Foundation Trust
- ◯ The Shrewsbury & Telford NHS Trust
- ◯ University Hospital Southampton NHS Foundation Trust
- ◯ Worcestershire Acute Hospitals NHS Trust
- ◯ Wrightington, Wigan & Leigh NHS Foundation Trust

2. Please select Job Role

- ◯ Consultant obstetrician
- ◯ Consultant anaesthetist
- ◯ Midwife

3. If applicable, please provide the grade or band of your current job role? (i.e. band 5)

Risk Assessment

4. Does your maternity unit perform a PPH risk assessment when a woman or birthing person is admitted in labour? *

- ◯ Yes
- ◯ No

5. *If yes, which member of staff typically completes the PPH risk assessment? (Please include band level if appropriate)*

6. Since implementing the OBS UK Care Bundle, has there been any change in time spent completing a PPH risk assessment compared to before implementation? *

- ◯ Yes
- ◯ No

7. *If yes, is this an increase or reduction in time spent per patient?*

- ◯ Increase
- ◯ Reduction

8. *How much has this time changed? (minutes)*

Proforma

9. Is your maternity unit using a version of the OBS UK proforma (paper or adapted electronic version) for contemporaneous PPH documentation (>500mL)? *

◯ Yes

◯ No

10. *If yes, is the proforma paper or electronic?*

◯ Paper

◯ Electronic

11. Which member of staff typically completes the PPH documentation? (Please include band level if appropriate) *

[ ]

12. Since implementing the OBS UK Care Bundle, has there been any change in time spent completing the PPH documentation compared to before implementation? *

◯ Yes

◯ No

13. If yes, is this an increase or reduction in time spent? *

◯ Increase

◯ Reduction

14. How much has this time changed? (minutes)

[ ]

Measured Blood Loss - birthing room

In this section, please describe the measured blood loss that takes place in a birthing room

15. Is your maternity unit typically measuring blood loss in all births that take place in birthing rooms? *

◯ Yes

◯ No

16. Which member of staff typically measures blood loss in this setting? (Please include band level if appropriate) *

17. Since implementing the OBS UK Care Bundle, has there been any change in time spent measuring and documenting blood loss compared to before implementation? *

◯ Yes

◯ No

18. *If yes, is this an increase or reduction in time spent?*

◯ Increase

◯ Reduction

19. How much has this time changed? (minutes)

Measured Blood Loss - theatre

In this section, please describe the measured blood loss that takes place in a theatre setting.

20. Is your maternity unit typically measuring blood loss in all births that take place in theatre? *

◯ Yes

◯ No

21. Which member of staff typically measures blood loss in this setting? (Please include band level if appropriate)

____

22. Since implementing the OBS UK Care Bundle, has there been any change in time spent measuring and documenting blood loss compared to before implementation? *

◯ Yes

◯ No

23. *If yes, is this an increase or reduction in time spent?*

◯ Increase

◯ Reduction"

24. How much has this time changed? (minutes)

____

Escalation and intervention – 500-999mL blood loss in a birthing room

In this section, please describe a patient who has lost 500-999mL in a birthing room (i.e. not in a theatre setting) in whom there is no concern of concealed bleeding or coagulopathy.

25. Since implementing the OBS UK Care Bundle, has there been any change in staff attendance during 500-999mL PPH compared to before implementation? *

- ◯ Yes
- ◯ No

26. *If yes, please select a member of staff who has been affected.*

- ◯ Midwife
- ◯ Obstetrician
- ◯ Anaesthetist
- ◯ Haematologist

27. *Please specify their band/grade.*

28. For the selected member of staff, is this an increase or a reduction in time spent managing the PPH?

- ◯ Increase
- ◯ Reduction

29. How much has this time changed? (minutes)

30. If applicable, please select the **second** member of staff who has been affected.

- ◯ Not applicable
- ◯ Midwife
- ◯ Obstetrician
- ◯ Anaesthetist
- ◯ Haematologist

31. Please specify their band/grade.

32. For the selected member of staff, is this an increase or a reduction in time spent managing the PPH?

- Increase
- Reduction

33. How much has this time changed? (minutes)

34. If applicable, please select the **third** member of staff who has been affected.

- Not applicable
- Midwife
- Obstetrician
- Anaesthetist
- Haematologist

35. Please specify their band/grade.

36. For the selected member of staff, is this an increase or a reduction in time spent managing the PPH?

- Increase
- Reduction

37. How much has this time changed? (minutes) *

38. If applicable, please select the **fourth** member of staff who has been affected.

- ◯ Not applicable
- ◯ Midwife
- ◯ Obstetrician
- ◯ Anaesthetist
- ◯ Haematologist

39. Please specify their band/grade.

[ ]

40. For the selected member of staff, is this an increase or a reduction in time spent managing the PPH?

- ◯ Increase
- ◯ Reduction

41. How much has this time changed? (minutes) *

[ ]

Escalation and intervention – 1000-1499mL blood loss in any setting

In this section, please describe a patient who has lost 1000-1499mL in any setting (i.e. a birthing room or theatre) or concerns of concealed bleeding or coagulopathy.

42. Since implementing the OBS UK Care Bundle, has there been any change in staff attendance during 1000-1499mL PPH compared to before implementation? *

- ◯ Yes
- ◯ No

43. If yes, please select a member of staff who has been affected. *

- ◯ Midwife
- ◯ Obstetrician
- ◯ Anaesthetist
- ◯ Haematologist

44. Please specify their band/grade.

[ ]

45. For the selected member of staff, is this an increase or a reduction in time spent managing the PPH?

- ◯ Increase
- ◯ Reduction

46. How much has this time changed? (minutes)

[ ]

47. If applicable, please select the **second** member of staff who has been affected.

- ◯ Not applicable
- ◯ Midwife
- ◯ Obstetrician
- ◯ Anaesthetist
- ◯ Haematologist

48. Please specify their band/grade.

49. For the selected member of staff, is this an increase or a reduction in time spent managing the PPH?

- ◯ Increase
- ◯ Reduction

50. How much has this time changed? (minutes)

51. If applicable, please select the **third** member of staff who has been affected.

- ◯ Not applicable
- ◯ Midwife
- ◯ Obstetrician
- ◯ Anaesthetist
- ◯ Haematologist

52. Please specify their band/grade.

53. For the selected member of staff, is this an increase or a reduction in time spent managing the PPH?

- ◯ Increase
- ◯ Reduction

54. How much has this time changed? (minutes)

55. If applicable, please select the **fourth** member of staff who has been affected.

- ◯ Not applicable
- ◯ Midwife
- ◯ Obstetrician
- ◯ Anaesthetist
- ◯ Haematologist

56. Please specify their band/grade.

[ ]

57. For the selected member of staff, is this an increase or a reduction in time spent managing the PPH?

- ◯ Increase
- ◯ Reduction

58. How much has this time changed? (minutes)

[ ]

59. Has there been any change in actions performed by the multiprofessional team during 1000-1499mL PPH compared to before implementation (e.g., time taken to undertake point-of-care blood tests)? *

- ◯ Yes
- ◯ No

60. If yes, please provide details if applicable

[ ]

Escalation and intervention – >/=1500mL blood loss and ongoing bleeding in any setting

In this section, please describe a patient who has lost 1500mL or more and has ongoing bleeding in any setting (i.e. a birthing room or theatre).

61. Since your unit implemented the OBS UK Care Bundle has there been any change in **staff attendance** during >/=1500mL PPH compared to before implementation? *

- ◯ Yes
- ◯ No

62. If yes, please select a member of staff who has been affected. *

- ◯ Midwife
- ◯ Obstetrician
- ◯ Anaesthetist
- ◯ Haematologist

63. Please specify their band/grade.

[ ]

64. For the selected member of staff, is this an increase or a reduction in time spent managing the PPH?

- ◯ Increase
- ◯ Reduction

65. How much has this time changed? (minutes) *

[ ]

66. If applicable, please select the **second** member of staff who has been affected. *

- ◯ Not applicable
- ◯ Midwife
- ◯ Obstetrician
- ◯ Anaesthetist
- ◯ Haematologist

67. Please specify their band/grade. *

68. For the selected member of staff, is this an increase or a reduction in time spent managing the PPH?

- ◯ Increase
- ◯ Reduction

69. How much has this time changed? (minutes)

70. If applicable, please select the **third** member of staff who has been affected.

- ◯ Not applicable
- ◯ Midwife
- ◯ Obstetrician
- ◯ Anaesthetist
- ◯ Haematologist

71. Please specify their band/grade.

72. For the selected member of staff, is this an increase or a reduction in time spent managing the PPH?

- ◯ Increase
- ◯ Reduction

73. How much has this time changed? (minutes)

74. If applicable, please select the **fourth** member of staff who has been affected.

- ◯ Not applicable
- ◯ Midwife
- ◯ Obstetrician
- ◯ Anaesthetist
- ◯ Haematologist

75. Please specify their band/grade.

[ ]

76. For the selected member of staff, is this an increase or a reduction in time spent managing the PPH?

- ◯ Increase
- ◯ Reduction

77. How much has this time changed? (minutes)

[ ]

78. Since your unit implemented the OBS UK Care Bundle has there been any change in **staff who are alerted** during >/=1500mL PPH compared to before implementation? *

- ◯ Yes
- ◯ No

79. If yes, please select a member of staff who has been alerted. *

- ◯ Midwife
- ◯ Obstetrician
- ◯ Anaesthetist
- ◯ Haematologist

80. Please specify their band/grade.

[ ]

81. For the selected member of staff, is this an increase or a reduction in time spent being alerted about the PPH?

- ◯ Increase
- ◯ Reduction

82. How much has this time changed? (minutes) *

83. If applicable, please select the **second** member of staff who has been alerted. *

- ◯ Not applicable
- ◯ Midwife
- ◯ Obstetrician
- ◯ Anaesthetist
- ◯ Haematologist

84. Please specify their band/grade. *

85. For the selected member of staff, is this an increase or a reduction in time spent managing the PPH?

- ◯ Increase
- ◯ Reduction

86. How much has this time changed? (minutes)

87. If applicable, please select the **third** member of staff who has been alerted.

- Not applicable
- Midwife
- Obstetrician
- Anaesthetist
- Haematologist

88. Please specify their band/grade.

89. For the selected member of staff, is this an increase or reduction in time spent being alerted about the PPH?

- Increase
- Reduction

90. How much has this time changed? (minutes)

91. If applicable, please select the **fourth** member of staff who has been alerted.

- Not applicable
- Midwife
- Obstetrician
- Anaesthetist
- Haematologist

92. Please specify their band/grade.

93. For the selected member of staff, is this an increase or reduction in time spent being alerted about the PPH?

- ◯ Increase
- ◯ Reduction

94. How much has this time changed? (minutes)

95. Since your unit implemented the OBS UK Care Bundle has there been any change in actions performed by the multi-professional team during >/=1500mL PPH compared to before implementation (e.g. time taken to undertake point of care blood tests)? *

- ◯ Yes
- ◯ No

96. *If yes - please provide details*

97. Please select a member of staff who has been affected.

- ◯ Midwife
- ◯ Obstetrician
- ◯ Anaesthetist
- ◯ Haematologist

98. Please specify their band/grade.

99. For the selected member of staff, is this an increase or a reduction in time spent?

- ◯ Increase
- ◯ Reduction

100. How much has this time changed? (minutes)

101. If applicable, please select the **second** member of staff who has been affected.

- ◯ Not applicable
- ◯ Midwife
- ◯ Obstetrician
- ◯ Anaesthetist
- ◯ Haematologist

102. Please specify their band/grade.

[ ]

103. For the selected member of staff, is this an increase or a reduction in time spent?

- ◯ Increase
- ◯ Reduction

104. How much has this time changed? (minutes)

[ ]

105. If applicable, please select the **third** member of staff who has been affected.

- ◯ Not applicable
- ◯ Midwife
- ◯ Obstetrician
- ◯ Anaesthetist
- ◯ Haematologist

106. Please specify their band/grade.

[ ]

107. For the selected member of staff, is this an increase or reduction in time spent?

- ◯ "Increase
- ◯ Reduction"

108. How much has this time changed? (minutes)

____

109. If applicable, please select the **fourth** member of staff who has been affected.

- ○ Not applicable
- ○ Midwife
- ○ Obstetrician
- ○ Anaesthetist
- ○ Haematologist

110. Please specify their band/grade.

____

111. For the selected member of staff, is this an increase or reduction in time spent?

- ○ "Increase
- ○ Reduction"

112. How much has this time changed? (minutes)

____

This content is neither created nor endorsed by Microsoft. The data you submit will be sent to the form owner.

Microsoft Forms
