# Supplementary material for: Obstetric Bleeding Study UK (OBS UK): protocol for a stepped wedge cluster randomised trial investigating the clinical and cost-effectiveness of a maternity quality improvement programme to reduce excess bleeding and need for transfusion after childbirth
Source: BMJ Open. 2026 Aug 20;16(8):e118723. doi: 10.1136/bmjopen-2026-118723 (PMC13504888; doi:10.1136/bmjopen-2026-118723)
Supplement: Supplementary file 3 [file bmjopen-2026-118723.reviewer_comments.pdf]

PEER REVIEW HISTORY

BMJ Open publishes all reviews undertaken for accepted manuscripts. Reviewers are asked to complete a checklist review form and are provided with free text boxes to elaborate on their assessment. These free text comments are reproduced below.

ARTICLE DETAILS

Title (Provisional)

The Obstetric Bleeding Study UK (OBS UK): protocol for a stepped wedge cluster randomised trial investigating the clinical and cost-effectiveness of a maternity quality improvement programme to reduce excess bleeding and need for transfusion after childbirth.

Authors

Kotecha, Sarah Joanne; Potter, Claire; Hope-Bell, Josh; Riddell, Nathan S; Munnery, Kim; Onyimadu, Olu; Liberty, Catherine; Taylor, Hazel; Dop, Caitlin; De Lloyd, Lucy; parry-smith, william; Townson, Julia; Pallmann, Philip; Moody, Gwenllian; Moriarty, Yvonne; Deere, Rachel; Barlow, Christian; Dhadda, Amrit; Elsmore, Amy; Wilson, Seren; Millward, Helen; Siddall, Kate; Sanders, Julia; Stanworth, Simon J; Black, Mairead; Petrou, Stavros; Rai, Tanvi; Slade, Pauline; Hinton, Lisa; Fye, Haddy; Gur Geden, Ayse; Collis, Rachel E; Collins, Peter; Bell, Sarah

---

VERSION 1 - REVIEW

**Reviewer**	**1**
**Name**	**Dang, Shaonong**
**Affiliation**	**Xi’an Jiaotong University Health Science Center**
**Date**	**14-May-2026**
**COI**	**None**

Postpartum haemorrhage (PPH) is one of the important complications although the incident of PPH has been decreasing. This work tries to evaluate effect of intervention measures (OBS UK PPH care bundle) on reducing number of women who receive an allogenic RBC transfusion for PPH by using SW-CRT design, which could be more operational in practice. The following issues should be addressed further i possible.

1. Authors are suggested to present implementation time,i.e. when to begin, if possible.

2. If possible, it would be better to show features of all maternity units, especially after randomized allocation.

3. Authors are suggested to show potential limitations of this study, especially from study design.

4. it would be better to present some results if authors have.

**Reviewer**	**2**
**Name**	**Hamm, Rebecca**
**Affiliation**	**University of Pennsylvania, department of obstetrics and gynecology**
**Date**	**22-May-2026**
**COI**	**None**

This paper describes the protocol for an ambitious and exciting stepped wedge cluster randomized trial to evaluate a QI program around obstetric hemorrhage. It is a well written protocol paper, describing an important trial. A few suggestions prior to publication:

1. The paper would benefit from a clearer description of the anticipated mechanism of effect of this intervention on the outcomes selected. Specifically, this could help provide justification for transfusion as the primary outcome, as it seems that some of these interventions actually have the potential to increase transfusion rates. Also, given transfusion is additionally a lifesaving procedure, justification is warranted as to why reducing it is the primary goal (rather than severe PPH, ICU admission, other SMM, etc). A mechanism figure could be helpful here.

2. Related to the above comment, by instituting QBL for all deliveries, there is potential for ascertainment bias in regards to the blood loss outcomes. The implications of this should be discussed.

3. Missingness in the primary study question should be addressed.

VERSION 1 - AUTHOR RESPONSE

Reviewer: 1
Comments to the Author:

Postpartum haemorrhage (PPH) is one of the important complications although the incident of PPH has been decreasing. This work tries to evaluate effect of intervention measures (OBS UK PPH care bundle) on reducing number of women who receive an allogenic RBC transfusion for PPH by using SW-CRT design, which could be more operational in practice.

The following issues should be addressed further i possible.
1. Authors are suggested to present implementation time,i.e. when to begin, if possible.

We have now added study and implementation dates to page 4.

2. If possible, it would be better to show features of all maternity units, especially after randomized allocation.

Thank you, we will describe the features of the maternity units in the publication of the findings.

3. Authors are suggested to show potential limitations of this study, especially from study design.

We have now described the limitations in more detail on page 11.

4. it would be better to present some results if authors have.
We thank the reviewer for their interest in the study results. In line with BMJ Open publication policy we are submitting this protocol before data collection is complete and therefore have not conducted data analysis yet.

Reviewer: 2
Comments to the Author:

This paper describes the protocol for an ambitious and exciting stepped wedge cluster randomized trial to evaluate a QI program around obstetric hemorrhage. It is a well written protocol paper, describing an important trial. A few suggestions prior to publication:

1. The paper would benefit from a clearer description of the anticipated mechanism of effect of this intervention on the outcomes selected. Specifically, this could help provide justification for transfusion as the primary outcome, as it seems that some of these interventions actually have the potential to increase transfusion rates. Also, given transfusion is additionally a lifesaving procedure, justification is warranted as to why reducing it is the primary goal (rather than severe PPH, ICU admission, other SMM, etc). A mechanism figure could be helpful here.

Thank you, we have included the logic model for the intervention as a supplement and referred to it on pages 3 and 5.

2. Related to the above comment, by instituting QBL for all deliveries, there is potential for ascertainment bias in regards to the blood loss outcomes. The implications of this should be discussed.

We have now included this on page 11, in the discussion section.

3. Missingness in the primary study question should be addressed.

We have now included this on page 8.

VERSION 2 - REVIEW

**Reviewer**	**2**
**Name**	**Hamm, Rebecca**
**Affiliation**	**University of Pennsylvania, department of obstetrics and gynecology**
**Date**	**03-Aug-2026**
**COI**	

The authors have made appropriate edits to the manuscript and I now recommend it for publication.
