# Supplementary material for: Obstetric Bleeding Study UK (OBS UK): protocol for a stepped wedge cluster randomised trial investigating the clinical and cost-effectiveness of a maternity quality improvement programme to reduce excess bleeding and need for transfusion after childbirth
Source: BMJ Open. 2026 Aug 20;16(8):e118723. doi: 10.1136/bmjopen-2026-118723 (PMC13504888; doi:10.1136/bmjopen-2026-118723)
Supplement: Supplementary file 4 [file bmjopen-16-8-s001.docx]

**Supplemental Material**

**Supplement 1 - Terminology**

*The terms **woman/women/mother/breast feeding** are used consistently throughout this manuscript pertaining to the primary person receiving care and giving birth. We acknowledge that not all birthing people use these terms and we want to promote gender equality throughout to ensure respect for the unique psychological, physiological, and social needs of every individual. We specifically acknowledge the trans and non-binary experience of pregnancy and PPH and ensured to engage with people with this experience in a respectful, safe, and inclusive way.

The **birth partner** is the person who is present at the time of the birth and postpartum haemorrhage PPH, supporting the woman or birthing person. The **economic partner** is defined as the person who lives or contributes financially with the woman or birthing person who has given birth and this may be the birth partner, a spouse, a relative such as a parent or other individual.

**Maternities** describe the number of women giving birth (including live and stillbirth) in maternity services, rather than the number of babies born.

**Births** include all registerable births (any baby born live (at any gestation) or a stillbirth/termination of pregnancy if this occurs at or after 24^+0^ weeks of gestation).

**Supplement 2 - OBS UK Postpartum Haemorrhage (PPH) care bundle**

1) **Risk assessment:** Where practical a 'risk assessment' will identify a standard set of risk factors for PPH (based on RCOG guidance) and will categorise the level of risk accordingly. It will also include a set of triggers for action based upon the estimated level of risk. The risk assessment will be undertaken on arrival to an obstetric-led delivery unit or when a woman is in labour. This should be re-evaluated whenever a change in circumstances occurs as the risk may change over time.
2) **Quantification of blood loss (QBL):** Where practical, cumulative blood loss will be measured objectively from the time of birth in all women at the maternity unit using a validated method that combines volumetric and gravimetric measurements. Volumetric measurements will be derived from under-buttock drapes, drains and suction containers after amniotic fluid has been excluded. For gravimetric measurements, blood on pads swabs, or bedding will be measured by weighing and subtracting the known dry weight to give a blood loss in mL (1g=1mL). In the case of vaginal birth, the under-buttock pad is removed immediately after birth to accommodate amniotic fluid and then any further fluid is included in the blood loss measurement.
3) **Escalation of multi-professional care to more senior staff at defined volumes of blood loss (or earlier if low maternal weight/BMI) with appropriate medical intervention:**
a) By 500 mL the senior clinical midwife is informed. If the mother is not in an obstetric unit and bleeding is ongoing, consider transfer to an obstetric unit if this has not already happened
b) By 1 L, the senior midwife, an obstetrician and anaesthetist must attend the bedside to diagnose and treat the likely cause of bleeding if this has not already happened. If the woman is not in an obstetric unit, transfer to an obstetric unit should occur.
c) By 1.5 L, the consultant obstetrician and anaesthetist should be informed and attend if bleeding is ongoing if this has not already happened.
4) **Point-of-care test of coagulation, blood component replacement and tranexamic acid**

At 1 L blood loss with ongoing bleeding a point-of-care test of coagulation should be performed and a coagulation screen sent to the laboratory for routine testing. Coagulation tests may be undertaken earlier at the discretion of the treating clinicians for any clinical concern, for example concern of haemostatic impairment, placental abruption, amniotic fluid embolism or concealed bleeding. The point-of-care coagulation tests will be performed using either a ROTEM or TEG depending on which device the maternity unit has been allocated to.

The point-of-care coagulation test should be interpreted according to the OBS UK point-of-care algorithm. The algorithm identifies reduced levels of fibrinogen and promotes early replacement with a concentrated source of fibrinogen (either fibrinogen concentrate or cryoprecipitate). If the local maternity unit decides to use fibrinogen concentrate, any brand of fibrinogen concentrate may be used. A maternity unit may switch brand of fibrinogen concentrate during the study and may switch from cryoprecipitate to fibrinogen concentrate or vice versa at any time during the study. If deemed appropriate by the local multi-professional team, fibrinogen concentrate and cryoprecipitate may both be administered to an individual. The maternity unit should inform the study team if the source of concentrated fibrinogen changes and report the date this occurred.

The point-of-care test of coagulation should be repeated after each 500 mL additional blood loss, after infusion of fibrinogen concentrate, cryoprecipitate or fresh frozen plasma or at any time for clinical concern. The OBS UK algorithm should be used to interpret these tests and guide blood component infusion if indicated. The algorithm may not be applicable for women with inherited bleeding disorders, or who are fully anticoagulated and the local team should develop an individualised delivery plan. The algorithm may not be applicable in cases of coagulopathy due to sepsis.

Tranexamic acid 1g IV should be given as soon as abnormal bleeding is identified according to local protocols but must be given at the latest after 1L measured blood loss with ongoing bleeding. Tranexamic acid 1g IV should be repeated after 30 minutes if bleeding is ongoing.

Maternity units will be trained in the use, maintenance and quality control of the ROTEM or TEG devices by the manufacturer in conjunction with local point-of-care management teams. The national study team will train local personnel on the interpretation of the OBS UK algorithm, and this training will be cascaded to all relevant personnel.

**Supplement 3 – OBS UK Logic Model**

**Supplement 4 – OBS UK Quality Improvement (QI) Programme**

A national multiprofessional study team will support local maternity units by organising and leading initiation meetings, providing training and care bundle resources to maternity units, supporting units with local QI data collection and interpretation, supporting units in adopting OBS UK PPH care (cascading educational events, providing training and education materials), creating an enthusiasm for change and working with local leaders to remove barriers to change and facilitating networks between units to accelerate learning and adoption.

The local QI champion teams will receive training in QI methodology and skills through the online initiation meetings, enabling them to interpret process and outcome data and achieve change. The local QI team will integrate training into local training opportunities including multi-professional simulation training, ad hoc sessions and monthly QI/audit sessions. Plan-do-study-act (PDSA) cycles will include measures of frequency of training, attendance, and feedback to ensure standardisation and uptake of the intervention.

At months 1, 4 and 7 of the 9-month implementation period the local QI team will conduct audits of 30 reasonably consecutive deliveries (all locations, not just obstetric unit births), irrespective of blood loss, and undertake case note reviews of 10 reasonably consecutive women who had experienced an obstetric haemorrhage of more than 1L. The audit will establish the proportion of women who have had a risk assessment performed and had QBL after birth. The case note reviews will identify the proportion of women with PPH who have a completed risk assessment in their notes, had QBL from the time of birth, evidence of completion of OBS UK standardised paperwork, evidence for multi-professional attendance at 1L blood loss or clinical concern and whether point-of-care tests of coagulation were performed and appropriately interpreted using the OBS UK algorithm. The results of the audit and case note reviews and comparison with other anonymised maternity units will be fed back to staff on the maternity unit by the local QI champion team to inform areas for change.

**Supplement 5 - OBS UK Trial Monitoring Plan**

| **Metric** | **Frequency** |
| --- | --- |
| Birth and number of maternities and number of RBC transfusions overall | Monthly  6 monthly (TSC/IDMEC) |
| Birth and number of maternities per site | Monthly |
| List of sites using usual care and list of sites using OBS UK care | Monthly |
| Non-compliances per site. Non compliances for the bundle will not be reported but non-compliance with other procedures will be reported | Monthly |
| Optouts and withdrawals overall | Monthly  6 monthly (TSC/IDMEC) |
| Optouts and withdrawals per site | Monthly |
| Data upload completed overall | Monthly  6 monthly (TSC/IDMEC) |
| Data upload completed per site | Monthly |
| Recruitment to sub-studies overall | Monthly  6 monthly (TSC/IDMEC) |
| Recruitment to sub-studies by sites | Monthly |
| Sub study data queries | Monthly |
